# Editorial: Cytokines and CNS diseases

**DOI:** 10.3389/fncel.2023.1335455

**Published:** 2023-11-27

**Authors:** Roumen Balabanov

**Affiliations:** Department of Neurology, Feinberg School of Medicine, Northwestern University, Chicago, IL, United States

**Keywords:** cytokines, central nervous system, inflammation, autoimmune disease, neurodegenera-tion, autoimmune epilepsy

Cytokines are signaling molecules regulating the immune system function in health and disease (Dinarello, 2007). They represent a special class of soluble proteins that are produced by immune and non-immune cells in response to tissue injury or infection, which in turn exert local and systemic immunological as well as other biological effects. Cytokines are organized in a network of inter-cellular interactions connecting the immune system and its targets, facilitating immune activation, tissue inflammation or repair. They are commonly classified based on specific cell function such as antiviral interference (interferons), tumor cell cytotoxicity (tumor necrosis factors), inter-leukocyte communications (interleukins, chemokines), or hematopoietic stem cell proliferation (colony-stimulating factors). In the context of immunoregulation and autoimmune diseases, cytokines can play either pro-inflammatory and anti-inflammatory role, mediate a particular T and B cell response, or a disease process. Because of their protein nature, relatively small molecular weight and minute concentration in the blood, cytokines can be studied analytically and targeted therapeutically (Saxton et al., 2023).

The role of cytokines in the central nervous system (CNS) and neurological diseases remains understudied. Nevertheless, numerous studies have demonstrated that cytokines are important in the regulation of CNS homeostasis, neuronal development and synaptogenesis and they are intimately involved in the pathogenesis of epilepsy, autoimmune, and neurodegenerative disorders (Boulanger, 2009; Becher et al., 2017). Resident glial cells emerged as a potent source of cytokines modulating local microenvironment, synaptic transmission, and neuronal function. The evidence that locally produced cytokines can play an immunoregulatory role in CNS inflammation led to re-interpretation of the immune privilege concept of the CNS and the outside-in inflammatory hypothesis of CNS autoimmunity. Theoretically, the hypothesis of neuroimmune network founded by pleotropic cytokines signaling, allows for a more dynamic interpretation of neuroinflammation as well as for identification of cytokine-based therapeutic targets. Introduction of interferon-beta and anti-interleukin 6 (IL-6) or anti-tumor necrosis factor-alpha (TNF-α) therapies in clinical practice advanced the field of autoimmune neurology and provided a direct support for the critical involvement of cytokines in CNS diseases (Azodi and Jacobson, 2016).

The present Research Topic contains publications that are focused on novel and clinically relevant aspects of the cytokine role in CNS diseases. Grebenciucova and VanHaerents review the normal physiological role of IL-6, and its involvement in neuroinflammatory, autoimmune and SARS-CoV2 -associated disorders, and discuss the clinical utility of anti-IL-6 therapies. Basnyat et al. report on a positive association between IL-6 plasma levels and anti- glutamic acid decarboxylase antibodies in patients with autoimmune epilepsies, providing additional evidence for the involvement of this cytokine in this type of disorders and the therapeutic significance of IL-6 targeting. Caldito provides a broad review on the role of TNF-α in neuroinflammation and neuromodulation and highlights the differential role of its receptors in demyelinating disorders, and the paradoxical effects of anti-TNF-α therapies. Abbaoui et al. discuss the role of meningeal T cells in neurodegenerative disorders and the effect of T cells cytokines in modulating neuronal function and behavior. Finally, Giraldo et al. report on the immunoregulatory role of granulocyte-colony stimulating factor (G-CSF) that was successfully used in the setting of co-existent autoimmune disorder and immunodeficiency. This Research Topic in our opinion, though not comprehensive and limited in its scope, does highlight the important themes in the field of cytokines and CNS diseases and the directions of future research explorations.

## Author contributions

RB: Writing – original draft.
